# *Scutellaria baicalensis* exosome-like nanoparticles combat lung infection caused by *Mycoplasma gallisepticum* by regulating calcium homeostasis

**DOI:** 10.1186/s40104-026-01395-x

**Published:** 2026-05-05

**Authors:** Yecheng Yao, Ruiying Hu, Yifan Li, Mingyu Yu, Fangbing Xu, Yuquan Guo, Shun Wang, Liyang Guo, Jichang Li, Chunli Chen, Zhiyong Wu

**Affiliations:** 1 College of Veterinary Medicine, Northeast Agricultural University, 600 Changjiang Road, Xiangfang District, Harbin, 150030 People’s Republic of China; 2 Heilongjiang Key Laboratory for Animal Disease Control and Pharmaceutical Development, 600 Chang jiang Road, Xiang fang District, Harbin, 150030 People’s Republic of China

**Keywords:** Calcium ion homeostasis, Exosome-like nanoparticles, Inflammatory responses, miR159a, *Scutellaria baicalensis*

## Abstract

**Background:**

*Scutellaria baicalensis*, a traditional Chinese medicine (TCM), has demonstrated significant therapeutic efficacy in treating respiratory diseases caused by *Mycoplasma gallisepticum* (MG). However, the effective components of *Scutellaria baicalensis* are complex, and the material basis for its efficacy anti-MG infection remains unclear. This study aims to elucidate the molecular mechanism by which *Scutellaria baicalensis* exosome-like nanoparticles (SBELNs) and the key effector molecule, miR159a, regulate inflammation-induced injury caused by MG infection.

**Methods:**

SBELNs were isolated from *Scutellaria baicalensis* root by ultracentrifugation. The in vivo and in vitro transport of SBELNs was investigated through live imaging and laser confocal microscopy after staining with DIR fluorescent dye. Key miRNAs were screened via RNA sequencing, and target genes were predicted using online databases. The interaction between miR159a and its target gene, cyclic nucleotide-gated channel alpha 1 (*CNGA1*), was validated using a dual-luciferase reporter assay. Furthermore, the regulatory network of the miR159a/CNGA1 axis was systematically analyzed.

**Results:**

SBELNs can specifically target lung tissue. Subsequently, SBELNs release bioactive components that alleviate the lung inflammatory damage caused by MG infection. This beneficial effect stems from two aspects. Firstly, the flavonoid metabolites encapsulated in SBELNs directly suppress the inflammatory damage caused by MG infection. Secondly, the microRNA in SBELNs regulates calcium ion homeostasis via the miR159a/CNGA1 axis. This relieves the intracellular calcium overload induced by MG and participates in the regulation of the immune system by modulating calcium ions. The microRNA in SBELNs regulates calcium ion homeostasis through the miR159a/CNGA1 axis, thereby alleviating MG-induced intracellular calcium overload, mitochondrial damage, excessive ROS, and overactivation of the NF-κB inflammatory pathway.

**Conclusions:**

This article expounds that SBELNs alleviate lung injury caused by MG infection by regulating calcium homeostasis. This discovery demonstrates the anti-infective capability SBELNs, but also supports the development of natural drug delivery systems.

**Graphical Abstract:**

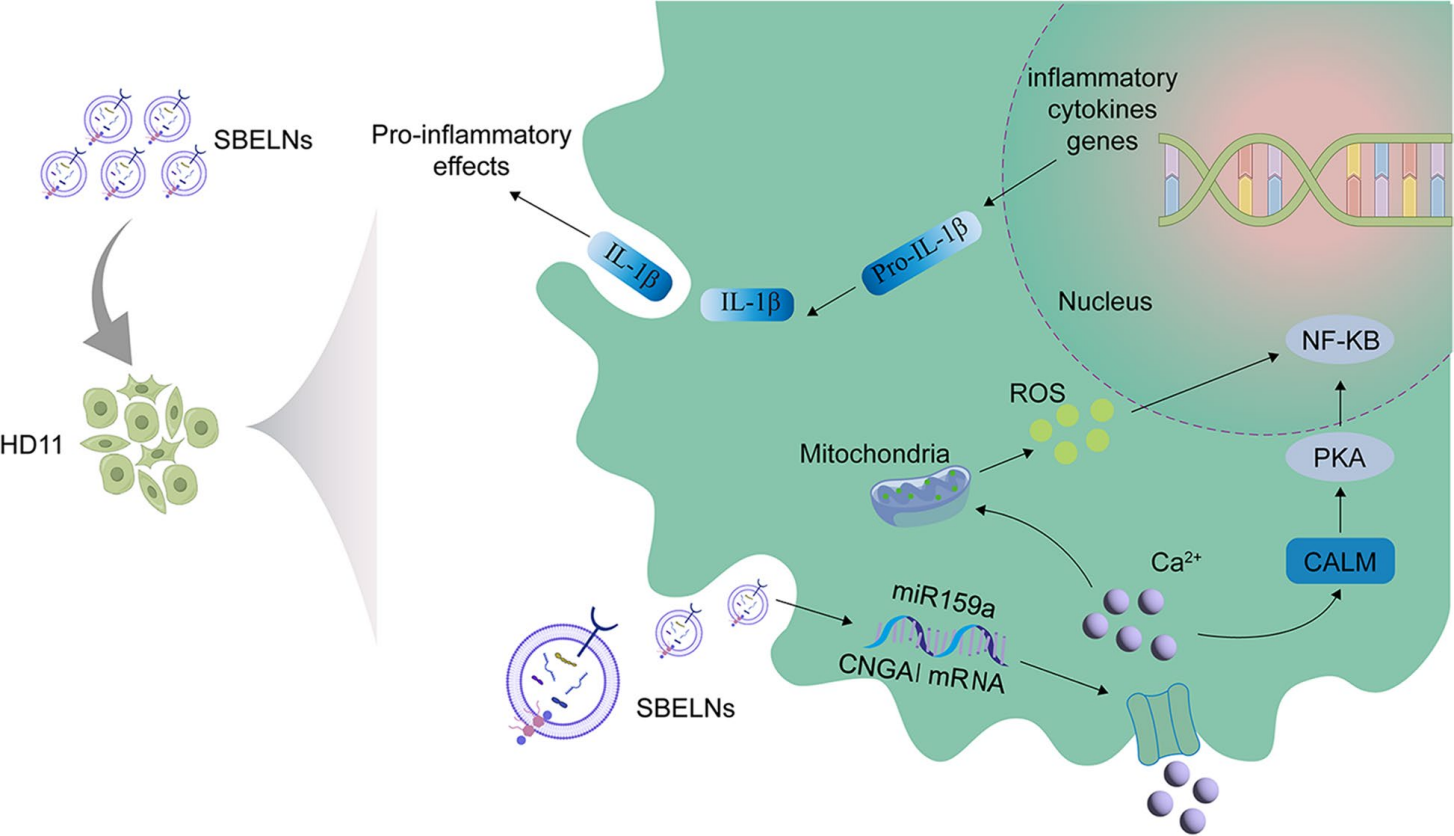

**Supplementary Information:**

The online version contains supplementary material available at 10.1186/s40104-026-01395-x.

## Introduction

*Mycoplasma gallisepticum *(MG), an avian pathogen lacking a cell wall and solely surrounded by a plasma membrane [1], poses a significant threat to the poultry industry. Once MG infects poultry flocks, it causes chronic respiratory disease, impairs humoral immunity, reduces growth performance, and leads to significant economic losses in poultry production [2]. At present, antibiotics are the primary means of managing MG infection, effectively inhibiting the colonization and proliferation of MG [3]. Nevertheless, the overuse of antibiotics has led to severe consequences, including increased MG resistance, antibiotic accumulation in poultry, and other associated problems [4]. As a result, the search for safe and effective alternative treatments has become a research focus. Due to its multi-component and multi-target nature, traditional Chinese medicine offers distinct advantages in the field of anti-infection, with *Scutellaria baicalensis* being a particularly notable subject of study [5].

The dried root of *Scutellaria baicalensis*, belonging to the Labiaceae family, is a common Chinese medicinal substance. The chemical composition of *Scutellaria baicalensis* is relatively intricate, and it has been discovered to encompass 57 chemical constituents [6]. Numerous studies have demonstrated that *Scutellaria baicalensis* exhibits favorable therapeutic effects on various diseases through its antiviral, anti-inflammatory, and antioxidant pharmacological properties [7]. Researchers have noticed that Baicalin has the potential to intervene MG-induced M1 activation of macrophages, thereby restoring the balance between M1 and M2 cells to a certain extent, consequently alleviating the respiratory mucosal injury and inflammatory damage caused by MG [8]. However, baicalin and baicalein, the main active components of *Scutellaria baicalensis*, are flavonoids and have a low bioavailability [9]. This suggests that after oral administration, these components may not be efficiently absorbed, potentially limiting their therapeutic efficacy. Exosome-like nanoparticles (ELNs), by contrast, are readily absorbed and represent a promising new material basis for the efficacy of TCM.

Plant-derived exosome-like nanoparticles (PDELNs) typically encompass lipids, nucleic acids, proteins and cellular metabolites [10]. PDELNs are frequently considered as mediators of intercellular signal transduction, which have excellent lipophilicity and can be effectively absorbed and utilized by animal organisms [11]. Recently, growing evidence has indicated that PDELNs possess excellent pharmacological properties, including anti-inflammatory, anti-tumor, and anti-fibrotic effects [12]. A recent study has shown that garlic ELNs can alleviate intestinal inflammation caused by sodium glucan sulfate by inhibiting the TLR4/MyD88/NF-κB signaling pathway, and can regulate intestinal microbes [13]. Chen et al. [14] have shown that ginger ELNs inhibit the assembly and activation of NLRP3 inflammasome and further inhibit downstream pathways, including caspase1 autolysis, IL-1β and IL-18 secretion, and pyroptotic cell death. These findings provide novel perspectives on the development and application of PDELNs. Nevertheless, the regulatory mechanism of SBELNs in MG infection remains elusive.

MicroRNA, a category of short non-coding RNAs, a significant portion of ELNs. A previous report showing that miRNA regulates many physiological activities of the body by targeting and silencing mRNA to control metabolism [15]. Plant-derived miRNAs from dietary sources have been shown to regulate animal metabolic pathways, reducing inflammation and offering therapeutic potential against multiple diseases [16]. Recently, research has demonstrated that *Catharanthus roseus *Don leaves-derived ELN regulate the host immune response by promoting the polarization and phagocytic activity of macrophages as well as the proliferation of lymphocytes [17]. Although accumulating evidence suggests that microRNAs carried by PDELNs hold significant therapeutic promise, the specific miRNA profiles of SBELNs and their regulatory mechanisms in MG pathogenesis remain largely unexplored.

This study demonstrated that SBELNs exhibit a remarkable tropism for lung tissue. Subsequently, they release bioactive components, thus mitigating the lung inflammatory damage triggered by MG infection. On the one hand, the flavonoid metabolites encapsulated in SBELNs directly suppress the inflammatory damage caused by MG infection. On the other hand, the microRNA in SBELNs regulates calcium ion homeostasis via the miR159a/CNGA1 axis. MiR159a directly targets the 3'UTR of *CNGA1* mRNA, suppressing CNGA1 protein expression and thereby inhibiting abnormal calcium channel activation. This process alleviates mitochondrial membrane potential damage, excessive ROS accumulation, and NF-κB pathway activation induced by MG infection. This discovery not only furnishes a theoretical foundation for the anti-MG application of SBELNs but also presents a novel viewpoint on the anti-infective property of TCM derived ELNs.

## Materials and method

### Isolation and characterization of SBELNs

The fresh root of *Scutellaria baicalensis* was added to five times the weight of distilled water, then evenly stirred at 500 × *g* for 3 h. The supernatant was taken and centrifuged at 5,000 × *g* for 0.5 h to remove debris and large organelles such as mitochondria and chloroplasts. The resulting supernatant was further centrifuged at 10,000 × *g* in a 4 °C centrifuge for 1 h, followed by filtration using a 0.22-μm filter membrane, and finally subjected to centrifugation at 180,000 × *g* in an ultra-high-speed centrifuge (Beckman Coulter, USA) at 4 °C for 1.5 h. The product of the centrifugation process was the enrichment area of SBELNs [18].

### Transmission electron microscopy (TEM) detection

Absorb 5 µL of sample onto the wax tray, place the copper mesh, and allow it to stand for 5 min. After drying, apply 1 drop of 3% phosphotungstic acid solution on the wax tray and fully contact the copper mesh for 4 min. The copper mesh was removed and dried under incandescent lamp. The SBELN morphology was observed by using TEM (HITACHI, Japan).

### Nanoparticle tracking analysis (NTA) detection

The particle size of SBELNs was measured using NTA (Malvern Panalytical, USA). After diluting 10 µL of the sample with 1 mL of PBS, a suitable sample pool was selected and slowly introduced into the particle size and potential analyzer (Zetasizer Nano ZS90) to avoid bubbles. Set the parameters of the NTA software (NTA 3.4) as follows: frame rate at 25 frames/s, 30 s duration, camera level at 9, laser selection Blue488, and measure each sample three times in repetition.

### Atomic force microscope detection

Take 20 µL of SBELNs diluted with ultrapure water and drop them onto freshly cleaved mica sheets, spread them evenly, and place them in a vacuum drying oven. After 3–4 h, use tweezers to pick up the dried sample mica sheets and carefully place them on the sample stage of the AFM (Brook, Malaysia). Then, calibrate the probe and set the parameters to take pictures and collect AFM images.

### Liquid chromatography tandem mass spectrometry (LC–MS/MS)

SBELNs were thawed at 4 °C and vortexed. 1 mL of the sample was taken into an EP tube and dried in a high-speed vacuum concentration centrifuge. Then, 1 mL of pre-cooled methanol–water (4:1, v/v) was added, followed by oscillatory mixing. The samples were placed in an automatic sampler at 4 °C and analyzed using an ultra-high performance liquid chromatography (Shimadzu Nexera X2) system with an ACQUITY UPLC HSS T3 column (2.1 mm × 100 mm, 1.8 µm; Waters, Milford, MA, USA). The injection volume was 4 µL, the column temperature was 40 °C, and the flow rate was 300 µL/min. The mobile phase A was 0.1% formic acid in water, and mobile phase B was 0.1% formic acid in acetonitrile. The gradient elution program was as follows: 0–1 min, 0% B; 1–5 min, B increased linearly from 0 to 48%; 5–6.4 min, B increased linearly from 48% to 100%; 6.4–8.4 min, B remained at 100%; 8.4–8.5 min, B decreased linearly from 100% to 0%; 8.5–10 min, B remained at 0%. Each sample was analyzed in both positive (+) and negative (–) ion modes using electrospray ionization (ESI). Mass spectrometry was performed on a TripleTOF^®^ 6600 mass spectrometer in positive and negative ion information-dependent acquisition mode. The mass spectrometry parameters were as follows: The instrument was set to acquire over the *m*/*z* range of 60–1,200 Da for TOF MS scan and 25–1,200 for product ion scan. The accumulation time for TOF MS scan was set at 0.08 s/spectrum and for product ion scan at 0.03 s/spectrum. Product ion scan was acquired using information-dependent acquisition with high sensitivity mode selected. In each cycle, 15 precursor ions were chosen for fragmentation at collision energy of 30 V with ± 15 spread. The ion source parameters are as follows: Positive ion mode: Source Temperature 550, Ion Source Gas1: 50, Ion Source Gas: 50, Curtain Gas: 50, Ion Spray Voltage Floating 5,500 V. Negative ion mode: Source Temperature 500, Ion Source Gas: 50, Ion Source Gas2: 50, Curtain Ga: 50, Ion Spray Voltage Floating (ISVF) −4,500 V.

### Labeling of SBELNs

SBELNs were labeled with Exosome Fluorescent labeling dye (DiR) (Umibio Science, China) through the following procedures: the DIR was diluted tenfold with 1 × PBS to prepare the dye working solution. The SBELNs were mixed with the dye working solution and stirred for 1 min using a vortex mixer. Subsequently, the mixture was incubated at 37 °C for 30 min. After incubation, 10 mL of 1 × PBS was added to the mixture, and the mixture was centrifuged at 4,500 × *g* for 15 min using an ultrafiltration tube to remove the excess dye. Finally, the extracellular vesicle precipitate was resuspended with 1 × PBS to obtain the SBELNs-DIR complex [19].

### Tracing of SBELNs

In vivo part: 1-day-old White Leghorn chickens were procured from Harbin Guangda Poultry Co., Ltd. in China and reared in the animal breeding facility. Twelve 7-day-old chickens were randomly divided into 4 groups: control group, SBELNs gavage for 12 h, 24 h, and 36 h groups, with 3 replicates in each group. The concentration of SBELNs-DIR complex was adjusted to 600 µg/mL for intragastric administration. The control group was given an equal volume of PBS solution by gavage. Each chick is given 0.2 mL of the SBELNs-DIR complex. Chickens were anesthetized with isoflurane. Following anesthesia, they were transferred to a small-animal live imaging chamber (Perkin Elmer, USA), and the imaging program was initiated to acquire images. To more intuitively explore the enrichment of SBELNs in vivo, after euthanasia, the chickens were dissected and the trachea, lungs, liver, spleen, intestines, and bursa of Fabricius were taken to collect images in the small-animal live imaging chamber to observe the distribution of SBELNs in tissues.

In vitro part: Well-grown Chicken-Like macrophages (HD-11) cells were digested with trypsin to make a cell suspension and inoculated in confocal cell culture dishes. After 8 h of culture, SBELNs-DIR complex (80 µg/mL) were added and co-incubated for 6, 12 and 24 h. After incubation, the small dishes were removed, the cells were washed with 1 × PBS, and 1 mL of 4% paraformaldehyde was added to fix the cells for 30 min at room temperature. After washing with 1 × PBS, 1 mL of 3% Triton X-100 was added to permeabilize the membrane for 15 min. After washing, 1 mL of 4',6-diamidino-2-phenylindole was added to stain the nuclei for 10 min at room temperature. The staining solution was discarded, and the cells were washed with 1 × PBS. The distribution of SBELNs and HD-11 cells was observed under a laser confocal microscope.

### Animals and drug

A total of 36 one-day-old chickens were randomly assigned to 4 groups (*n* = 9 per group): the normal control group (NC), the MG infection group (MG), the SBELNs intervention group (SBELNs), and the Tylosin treatment group (TYL). On the 7 days of age, chickens in the NC group were inoculated with 0.2 mL of MG medium into both sides of the air sacs. Chickens assigned to the MG, SBELNs, and TYL groups received bilateral air sac injections of 0.2 mL MG solution daily for 3 consecutive days. At 10 days of age, chickens in the SBELNs group were orally administered 1 mL/kg (chicken weight) of SBELNs solution (600 µg/mL) daily. Meanwhile, chickens in the TYL group started to be orally given TYL at a dose of 450 mg/kg/d (chicken weight) for 3 d. On the 14 days of age, 6 chickens were randomly selected from each group for humane euthanasia, and lung samples were collected.

### Histopathological examination

Fresh lung tissues were collected and fixed in 10% formalin for 48 h. After dehydration through different concentrations of alcohol gradients and clearing with xylene, the samples were embedded in paraffin and sectioned into 4-µm-thick slices. The slices were stained with hematoxylin–eosin (HE, Beyotime, China) and observed under an optical microscope.

### Immunofluorescence

After dewaxing the paraffin sections to water, antigen retrieval was carried out. A circle was traced around the tissue using a histochemical pen. The section was then placed in a 3% hydrogen peroxide solution and incubated at room temperature in the dark for 25 min. Serum was added for blocking and incubated for 30 min. After discarding the serum, the TNF-α primary antibody was added and incubated overnight at 4 °C. The fluorescent secondary antibody (Green) was added and incubated at room temperature for 50 min. IL-1β (Red) was labeled following the same procedure. The nuclei were counterstained with DAPI, and solution B for autofluorescence quenching was added and incubated for 5 min. The section was rinsed with running water for 10 min. An anti-fluorescence quenching mounting medium was added for mounting, and the section was observed under a fluorescence microscope.

### Cell viability assay

HD-11 cells were seeded in 96-well plates at a density of 1 × 10^5^ cells/well and cultured for 24 h. Subsequently, the cells were treated with different concentrations of SBELNs (0, 5, 10, 20, 40, 80 and 160 µg/mL) for 24 h. Cell viability was determined according to the instructions of the CCK-8 kit (Beyotime, China).

### Western blot (WB)

HD-11 cells were seeded in 6-well plates at a density of 1 × 10^5^ cells/well and cultivated for 24 h. Subsequently, the cells were incubated with 1 × PBS (control group) and SBELNs (80 µg/mL) respectively for 24 h. Furthermore, the cells were treated with MG (400 MOI) or 1 × PBS respectively for 24 h. Next, proteins were extracted from the cells by using a solution containing PMSF and RIPA, and the concentrations were subsequently determined with the BCA kit (Beyotime, China). Then, 5 × SDS up-sampling buffer was added and boiled for 10 min. Separation gels (10%) were utilized for the separation of proteins. The isolated proteins were transferred onto a nitrocellulose membrane and incubated in a rapid closure solution (Meilun Biotechnology Co., Ltd., Dalian, China) for 15 min. Subsequently, the samples were incubated at 4 °C overnight with a primary antibody, followed by incubation with the secondary antibody for 1 h at room temperature. The bound complexes were visualized through enhanced chemiluminescence reagents.

### RNA isolation and quantitative real-time polymerase chain reaction (qRT-PCR)

The total RNA was extracted from HD-11 cells by using TRIzol reagent (Life Science Technologies, USA). The RNA was transformed into cDNA with a reverse transcription kit (Takara, Beijing, China). The relative mRNA expression levels of target genes were quantified by qRT-PCR with the SYBR Premix Ex Taq kit (Takara, Beijing, China). *GAPDH* was employed as an internal reference gene.

### AO-EB kit detection

HD-11 cell damage was detected according to the instructions of the AO-EB detection kit (Leagene, China). In short, after different treatments, the HD-11 cells were added with AO-EB working solution. The results were then observed under a fluorescence microscope. In general, the stronger the red fluorescent light intensity of cells, the more serious the cell damage. The fluorescent light intensity was analyzed using the ImageJ software.

### SBELN RNA extraction and sequencing analysis

Total RNA was isolated from SBELNs using the TRIzol method. The concentration of sample RNA was determined with Nanodrop (Thermo Fisher, USA), and library quality was assessed with the Agilent 2100 Bioanalyzer instrument. Following successful quality inspection, a library was constructed and miRNA sequencing was conducted on the Illumina platform. Subsequently, the original data underwent de-jointing and mass filtering, followed by de-processing of the filtered sequences. The de-duplicated sequences were then compared against Rfan and miRBase databases to obtain annotation information for various RNAs. Furthermore, a detailed analysis of miRNAs was performed, including characterization and statistical analysis of miRNA expression levels.

### Prediction of miR159a target genes

Targeted analysis of the chicken genome for miR159a was conducted using the online website miRanda . Target genes with a sequence comparison score (sc) greater than 140 and a free energy (en) less than −30 were retained. The RNAhybrid online software was used to analyze the free energy of the target genes with higher scores in the Mirnada prediction results. Genes with a score less than −20 may have a targeting relationship. Select potential target genes by integrating the prediction results of the two methods [20].

### Luciferase reporter assay

The potential binding sites of miR159a and CNGA1-3′UTR wild type were predicted online using RNAhybrid. The mutant type site was obtained by site-directed mutagenesis. Using these two sequences as templates, approximately 430 bp sequences above and below them were amplified by PCR. The original sequence and the mutant sequence were respectively inserted into the pmirGlo luciferase vector to construct WT and MUT type luciferase expression vectors. The miR159a mimics and the pmirGlo luciferase expression vectors were co-transfected into 293 T cells using Lipofectamine™ LTX. The luciferase activity was detected 48 h after transfection using the Dual-LumiTM luciferase assay kit (TransGen Biotech, China).

### Fluo-4 calcium ion detection

Intracellular calcium ions were detected using the Fluo-4 calcium ion detection kit (Solarbio, China). HD-11 cells were seeded in 6-well plates at a density of 1 × 10^5^ cells/well and cultivated for 24 h. Subsequently, the cells were respectively incubated with 1 × PBS (control group) and miR159a mimics (20 μmol/L) for 24 h. Subsequently, the cells were respectively treated with MG (400 MOI) or 1 × PBS for 24 h. The results were then observed under a fluorescence microscope and statistically analyzed using the ImageJ software.

### Mitochondrial membrane potential detection

The mitochondrial membrane potential of cells was determined using the JC-1 detection kit (Solarbio, China). After subjecting the cells to the same treatment as mentioned above, the JC-1 working solution was added. Subsequently, the results were observed under a fluorescence microscope and statistically analyzed with the ImageJ software.

### Cell reactive oxygen species (ROS) assay

According to the instructions, ROS detection kit (Beyotime, China) was used to quantify the level of intracellular reactive oxygen species. After subjecting the cells to the same treatment as mentioned above, ROS working solution was added. The results were then observed under a fluorescence microscope and statistically analyzed using the ImageJ software.

### Statistical analysis

The data were analyzed with GraphPad Prism 10 (GraphPad, San Diego, CA, USA) and expressed as mean ± standard deviation (SD). Data were analyzed by one-way ANOVA using SPSS (version 24.0). Statistical differences were considered significant at ^*^P < 0.05, ^**^P < 0.01, or ^***^P < 0.001, and "ns" represents no significance.

## Result

### Extraction and identification of SBELNs

**Fig. 1:**
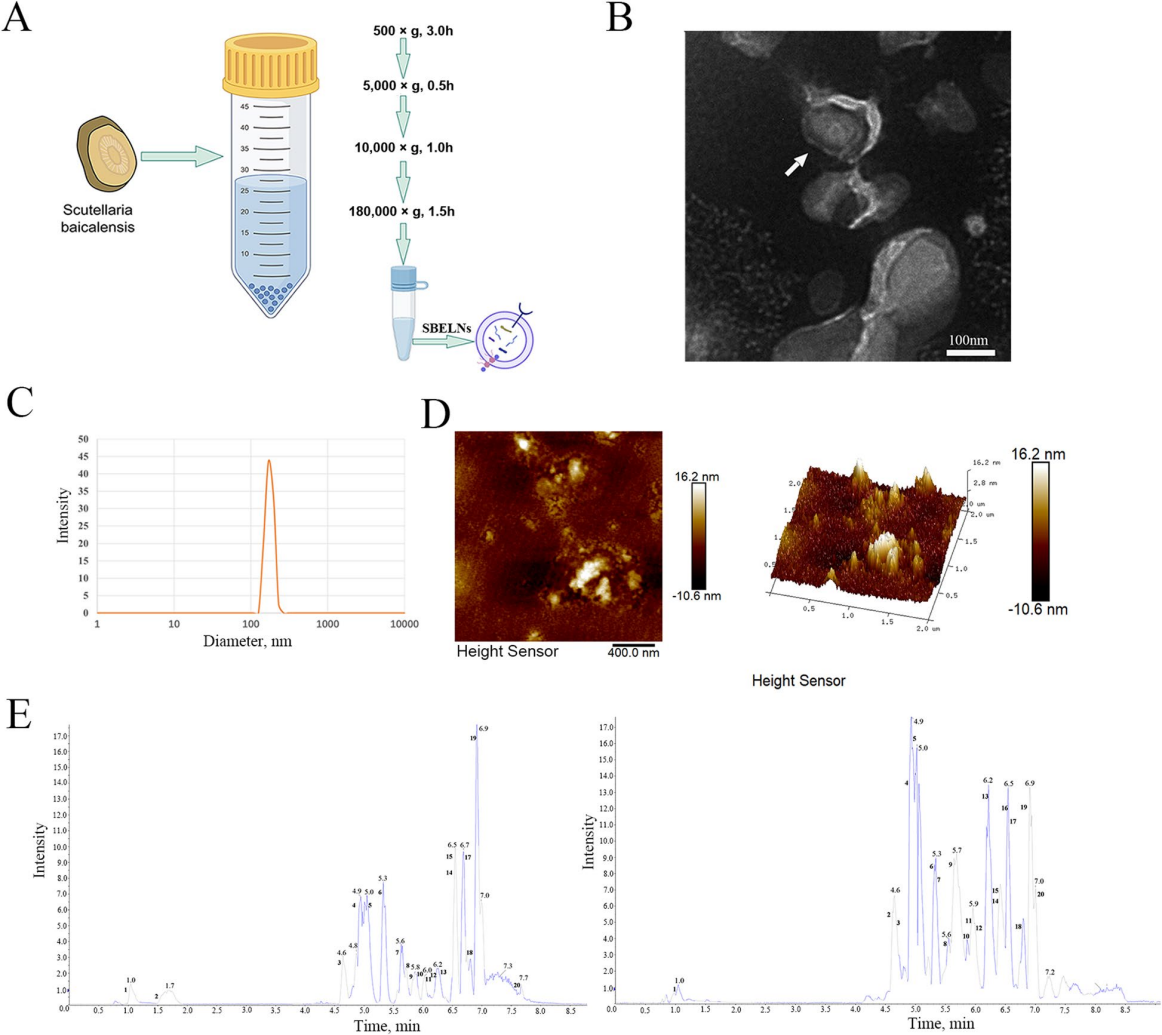
Extraction and identification of SBELNs. **A** Flowchart of SBELNs extraction. **B** Electron microscopy detection. **C** Nanoparticle microscopy detection. **D** Atomic force microscopy detection. **E** The detection of SBELNs by LC–MS/MS

To obtain the SBELNs, fresh *Scutellaria baicalensis* was ground into powder and the ELNs were extracted by ultracentrifugation (Fig. 1A). To identify the centrifugation products, TEM, NTA, and AFM were used to detect the SBELNs. TEM showed that the SBELNs were ellipsoidal structure (Fig. 1B). The diameter of the SBELNs detected by NTA ranged from 100 to 300 nm (Fig. 1C). AFM was used to detect the two-dimensional and three-dimensional structures of the SBELNs. It was found that the average height of the vesicles was approximately 16.2 nm and the particle size was around 200 nm (Fig. 1D). The LC–MS/MS detection results indicated that there were 52 components in SBELNs. The top 20 chemical components in both positive and negative ion flows were investigated, including sucrose, baicalin, wogonin, spiraeoside, corymboside, and so on (Fig. 1E and Tables 1 and 2). This was basically consistent with the detection by nanoparticle tracking analysis and conformed to the basic characteristics of PDELNs [21].

**Table 1:** The top 20 chemical components of SBELNs (Positive)

No.	RT, min	*m/z* (Negative)	Adduct	Formula	Metabolite name
1	1.04085	365.107	[M + Na]^+^	C_12_H_22_O_11_	Sucrose
2	1.695817	74.06062	[M + H]^+^	C_3_H_7_NO	3-Aminopropanal
3	4.6226	114.0918	[M + H]^+^	C_6_H_11_NO	epsilon-Caprolactam
4	4.934417	400.1497	[M + H]^+^	C_20_H_21_N_3_O_6_	Pelagiomicin A
5	5.09225	509.128	[M + H]^+^	C_23_H_24_O_13_	Limocitrin 3-glucoside
6	5.328217	274.0712	[M + H]^+^	C_14_H_11_NO_5_	Melandrin
7	5.5767	433.1122	[M + H]^+^	C_21_H_20_O_10_	Cosmosiin
8	5.7167	447.1265	[M + H]^+^	C_22_H_22_O_10_	Sissotrin
9	5.770367	300.0863	[M + H]^+^	C_16_H_13_NO_5_	Xanthevodine
10	5.85635	447.0998	[M + H]^+^	C_21_H_18_O_11_	Baicalin
11	5.87785	477.1048	[M + H]^+^	C_22_H_20_O_12_	6-O-Methylscutellarin
12	5.92085	285.0763	[M + H]^+^	C_16_H_12_O_5_	Oroxylin A
13	6.246167	287.0549	[M + H]^+^	C_15_H_10_O_6_	Kaempferol
14	6.528133	301.0713	[M + H]^+^	C_16_H_12_O_6_	Chrysoeriol
15	6.54965	271.0609	[M + H]^+^	C_15_H_10_O_5_	Baicalein
16	6.679133	253.047	[M + Na]^+^	C_13_H_10_O_4_	Visnagin
17	6.700467	274.2757	[M + H]^+^	C_16_H_35_NO_2_	Hexadecasphinganine
18	6.807967	413.0972	[M + Na]^+^	C_16_H_22_O_11_	Deacetylasperulosidic acid
19	6.91595	285.076	[M + H]^+^	C_16_H_12_O_5_	Wogonin
20	7.672733	149.0237	[M + H]^+^	C_8_H_4_O_3_	Phthalic anhydride

**Table 2:** The top 20 chemical components of SBELNs (Negative)

NO	RT, min	*m/z* (Negative)	Adduct	Formula	Metabolite name
1	1.044	377.0842	[M + Cl]^−^	C_12_H_22_O_11_	Melibiose
2	4.6499	463.0869	[M − H]^−^	C_21_H_20_O_12_	Spiraeoside
3	4.6714	563.1414	[M − H]^−^	C_26_H_28_O_14_	Corymboside
4	4.8444	207.0298	[M − H]^−^	C_10_H_8_O_5_	Fraxetin
5	4.9519	207.062	[M − H]^−^	C_11_H_12_O_4_	Sinapaldehyde
6	5.3288	144.0457	[M − H]^−^	C_9_H_7_NO	4-Hydroxyquinoline
7	5.4260	301.0364	[M − H]^−^	C_15_H_10_O_7_	Morin
8	5.5678	269.0451	[M −-H]^−^	C_15_H_10_O_5_	Apigenin
9	5.6868	177.0215	[M − H]^−^	C_9_H_6_O_4_	5,7-Dihydroxycoumarin
10	5.7841	461.1084	[M − H]^−^	C_22_H_22_O_11_	Termopsoside
11	5.9148	255.0299	[M − H]^−^	C_14_H_8_O_5_	Purpurin
12	5.9581	493.1311	[M + FA − H]^−^	C_22_H_24_O_10_	Isosakuranin
13	6.2196	213.0573	[M − H]^−^	C_13_H_10_O_3_	4,4'-Dimethylangelicin
14	6.4039	325.0333	[M − H]^−^	C_17_H_10_O_7_	Sophoracoumestan B
15	6.4039	285.0388	[M − H]^−^	C_15_H_10_O_6_	Luteolin
16	6.4149	423.034	[M − H]^−^	C_18_H_16_O_10_S	Mikanin 3-O-sulfate
17	6.5129	299.0565	[M − H]^−^	C_16_H_12_O_6_	Diosmetin
18	6.7856	537.0833	[M − H]^−^	C_30_H_18_O_10_	taiwaniaflavone
19	6.9166	373.0946	[M − H]^−^	C_19_H_18_O_8_	skullcapflavone II
20	6.9924	551.1	[M − H]^−^	C_31_H_20_O_10_	Isocryptomerin

### SBELN transport in vivo and in vitro

**Fig. 2:**
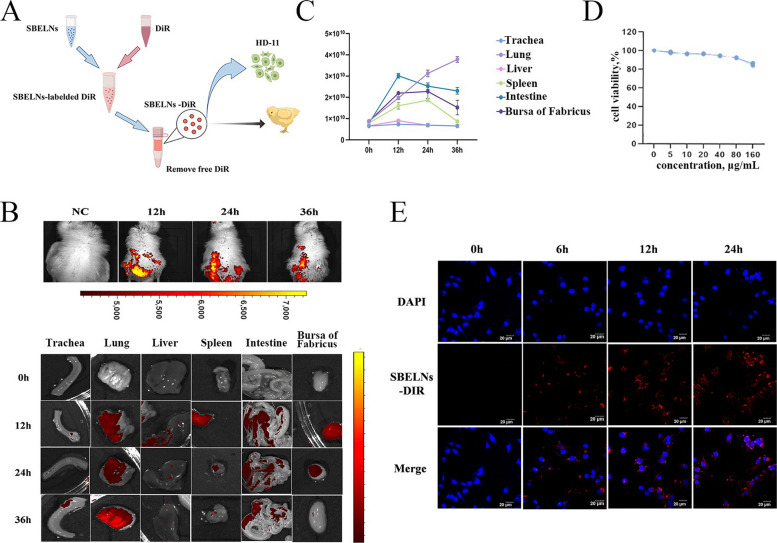
SBELN transport in vivo and in vitro. **A** SBELN staining and experimental procedures. **B** In vivo organ transport imaging of SBELNs: red indicates low concentration areas and yellow indicates high concentration areas. **C** Quantification of SBELNs in organs. **D** The cell viability of HD-11 was detected by CCK-8. **E** HD-11 cell uptake of SBELNs: the blue color represents the cell nuclei stained with DAPI, and the red color indicates the SBELNs-DIR complex

To explore the transport of SBELNs in vivo and in vitro, SBELNs were labeled with DIR fluorescent dye and administered the SBELNs-DIR complex to chickens by intragastric administration to observe its transport in the body. Subsequently, the SBELNs-DIR complex was co-incubated with HD-11 cells to investigate the phagocytic effect of the cells (Fig. 2A). In vivo, compared with the NC group, at 12 h, the SBELNs-DIR complex was mainly enriched in the gastrointestinal tract, and a small amount of the complex was present in some organs. With the passage of time, at 24 h and 36 h after intragastric administration, the SBELNs-DIR complex in the gastrointestinal tract was absorbed and transported to various organs of the body. To ascertain the distribution of SBELNs in organs, the chickens were dissected, the internal organs were extracted, and subsequently examined in the small-animal live imaging chamber. Compared with the NC group, at 12 h after intragastric administration, the SBELNs-DIR complex was mainly enriched near the intestine, and a small amount of the complex was present in the lungs, spleen, and bursa of Fabricius. At 24 h and 36 h after intragastric administration, the content of the SBELNs-DIR complex in the intestine, spleen, and bursa of Fabricius gradually decreased, while the concentration of the complex in the lungs gradually increased and reached a peak at 36 h (Fig. 2B and C). In vitro, the administration concentration of SBELNs detected by the CCK-8 kit was 80 µg/mL (Fig. 2D). In comparison to the NC group, a small quantity of the SBELNs-DIR complex was already detected within the HD-11 cells after 6 h of co-incubation. As incubation time progressed, following 12 and 24 h of co-incubation, the intensity of red fluorescence markedly increased, indicating substantial SBELNs-DIR complex is localized in the cytoplasm (Fig. 2E). In summary, after intragastric administration, SBELN was primarily absorbed through the gastrointestinal tract and transported to various organs via body fluids. SBELNs flowed into the lungs, spleen, and bursa of Fabricius. Over time, the SBELN complex eventually accumulated in the lungs. In vitro, SBELNs can also enter the HD-11 cell.

### SBELNs reduce lung tissue damage

**Fig. 3:**
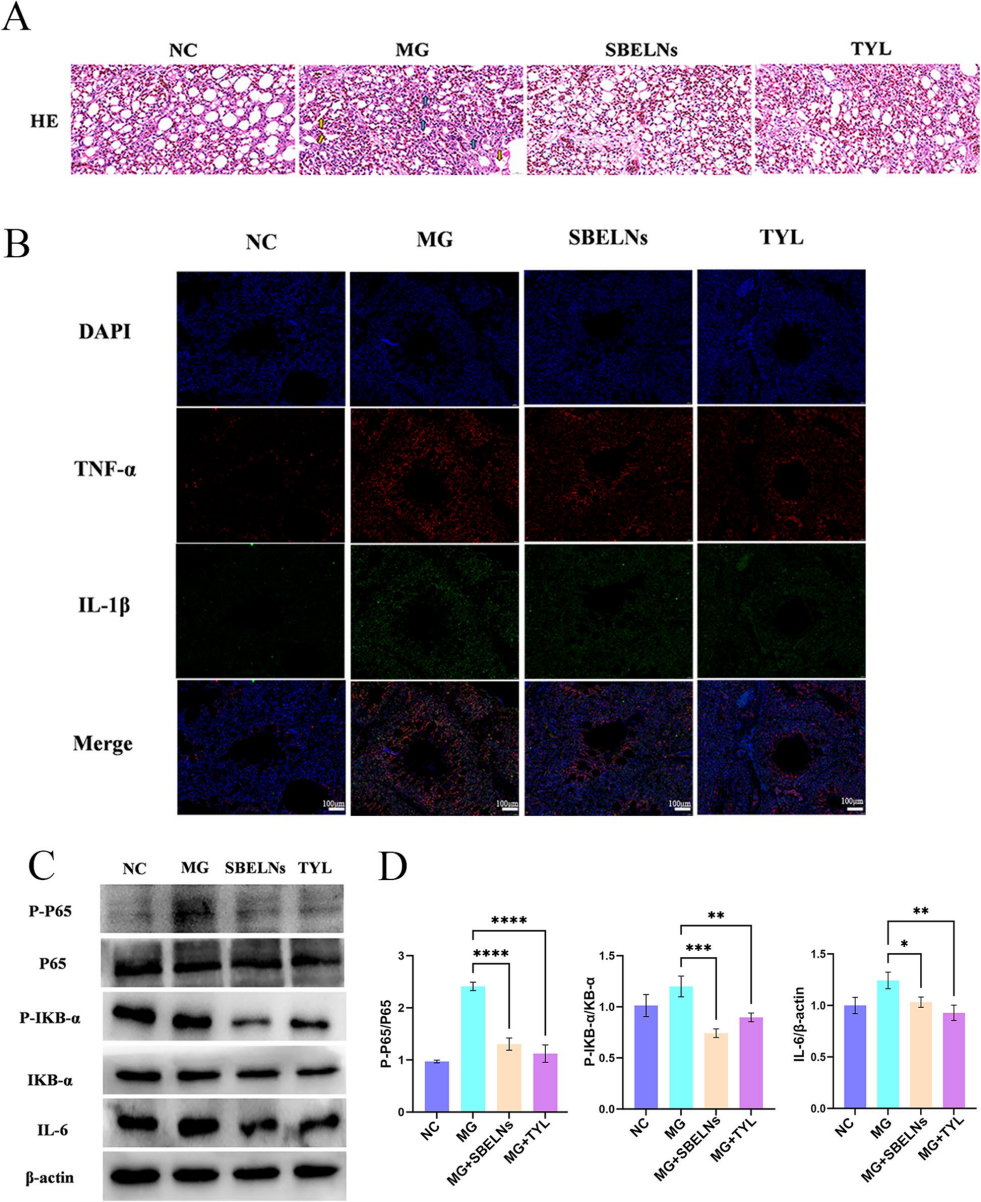
SBELNs reduce lung tissue damage. **A** HE was used to observe the pathological changes in lung tissue. The yellow arrows indicate alveolar rupture, and the blue arrows indicate inflammatory cell infiltration. **B** Immunofluorescence detection of inflammatory markers in lung tissue, DAPI (blue), TNF-α (red), IL-1β (green). **C**and** D** WB detection of inflammatory indicators and quantification. ^*^P < 0.05, ^**^P < 0.01, ^***^P < 0.001, ^****^P < 0.0001

To investigate the effects of SBELNs on the organism after they enter animals, a chicken model infected with MG was established. HE staining sections were employed to explore the histopathological alterations in the chickens. The HE staining results revealed that, in comparison with the control group, the alveolar walls in the MG-infected group were ruptured, and there were inflammatory exudates within the alveoli. These pathological manifestations were notably ameliorated following SBELNs treatment (Fig. 3A). Immunofluorescence and WB experiments were carried out to assess the inflammatory levels in the chickens. The immunofluorescence findings demonstrated that the levels of IL-1β and TNF-α in the lung tissue of the MG-infected group were significantly elevated, and were alleviated after SBELNs treatment (Fig. 3B). The WB results indicated that the levels of IL-6, P-IKB-α/IKB-α and P-p65/p65 in the lung tissue of the MG-infected group were markedly increased, and were decreased after SBELNs treatment (Fig. 3C and D). In summary, the alveolar structure disruption and inflammatory levels in the lungs of chickens were significantly exacerbated after MG infection, and were partially restored following SBELNs intervention.

### SBELNs alleviate cellular inflammatory damage

**Fig. 4:**
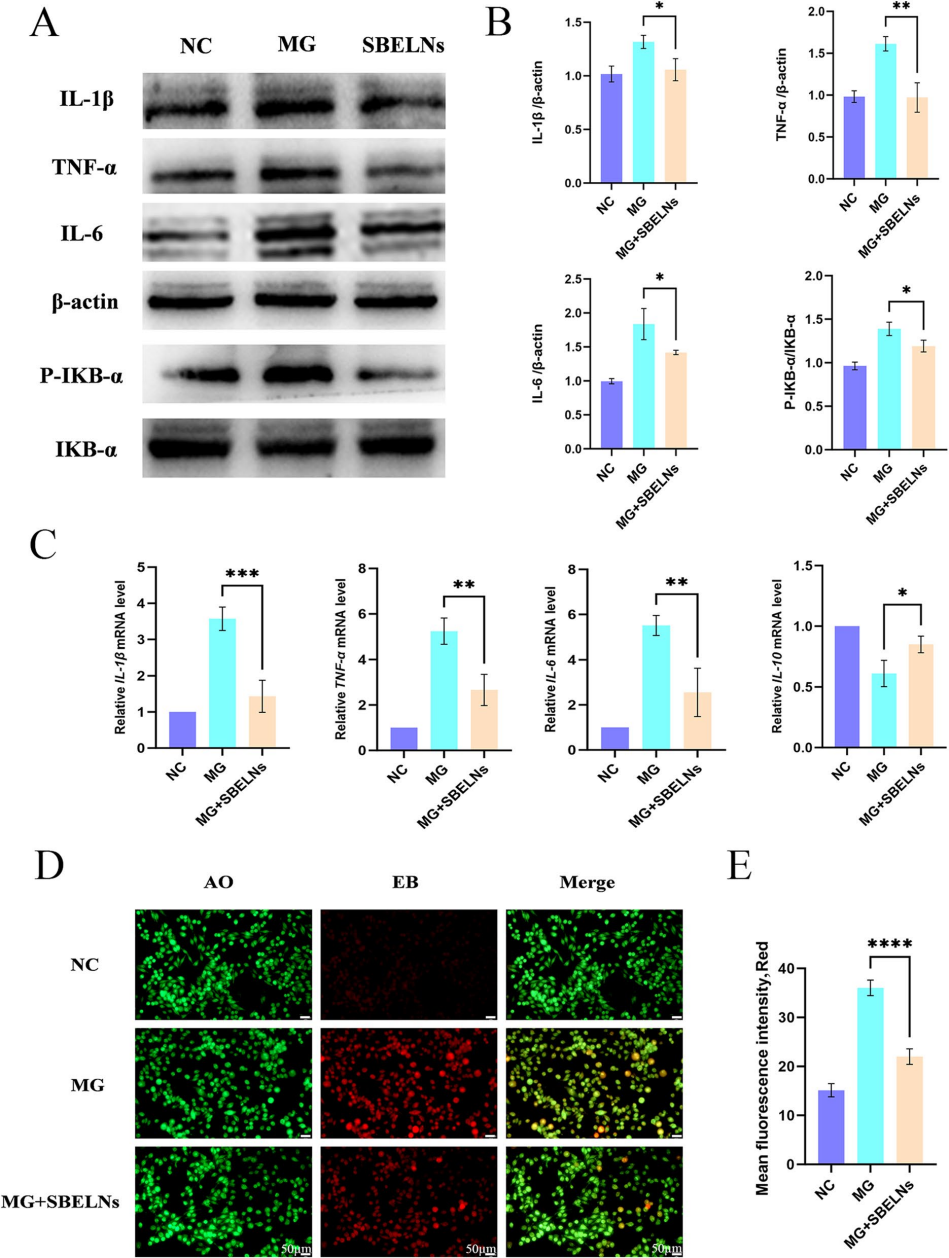
SBELNs alleviate cellular inflammatory injury. **A** and **B** WB detection of inflammatory indicators and quantification. **C** qRT-PCR detection of inflammatory indicators. **D** and **E** AO-EB kit detection of cellular injury: normal cells are green and spindle-shaped, while damaged cells are oval and pale yellow. ^*^P < 0.05, ^**^P < 0.01, ^***^P < 0.001, ^****^P < 0.0001

To investigate the regulatory role of SBELNs in MG-induced inflammatory responses, HD-11 cells were infected with MG and subsequently treated with SBELNs, after which changes in multiple inflammatory markers were assessed. The results of WB detection showed that after MG infection, the expression of pro-inflammatory factors such as *IL-1β*, *TNF-α*, *IL-6*, and P-IKB-α/IKB-α in HD-11 cells significantly increased, and the expression levels of inflammatory factors were partially restored after the addition of SBELNs (Fig. 4A and B). The results of qRT-PCR detection showed that after MG infection, the expression of pro-inflammatory factors such as *IL-1β*, *TNF-α*, and *IL-6* in HD-11 cells significantly increased, while the expression of the anti-inflammatory factor *IL-10* decreased. After the addition of SBELNs, the expression levels of inflammatory factors were partially restored (Fig. 4C). The results of AO-EB kit detection showed that after MG infection, the intensity of red fluorescence in cells significantly increased, and a large number of cells were damaged. After the addition of SBELNs, the intensity of red fluorescence partially recovered, and cell damage was alleviated (Fig. 4D and E). In conclusion, MG infection induces inflammatory damage to cells, while the addition of SBELNs mitigates such cellular injury.

### miR159a inhibits the expression of CNGA1 protein

**Fig. 5:**
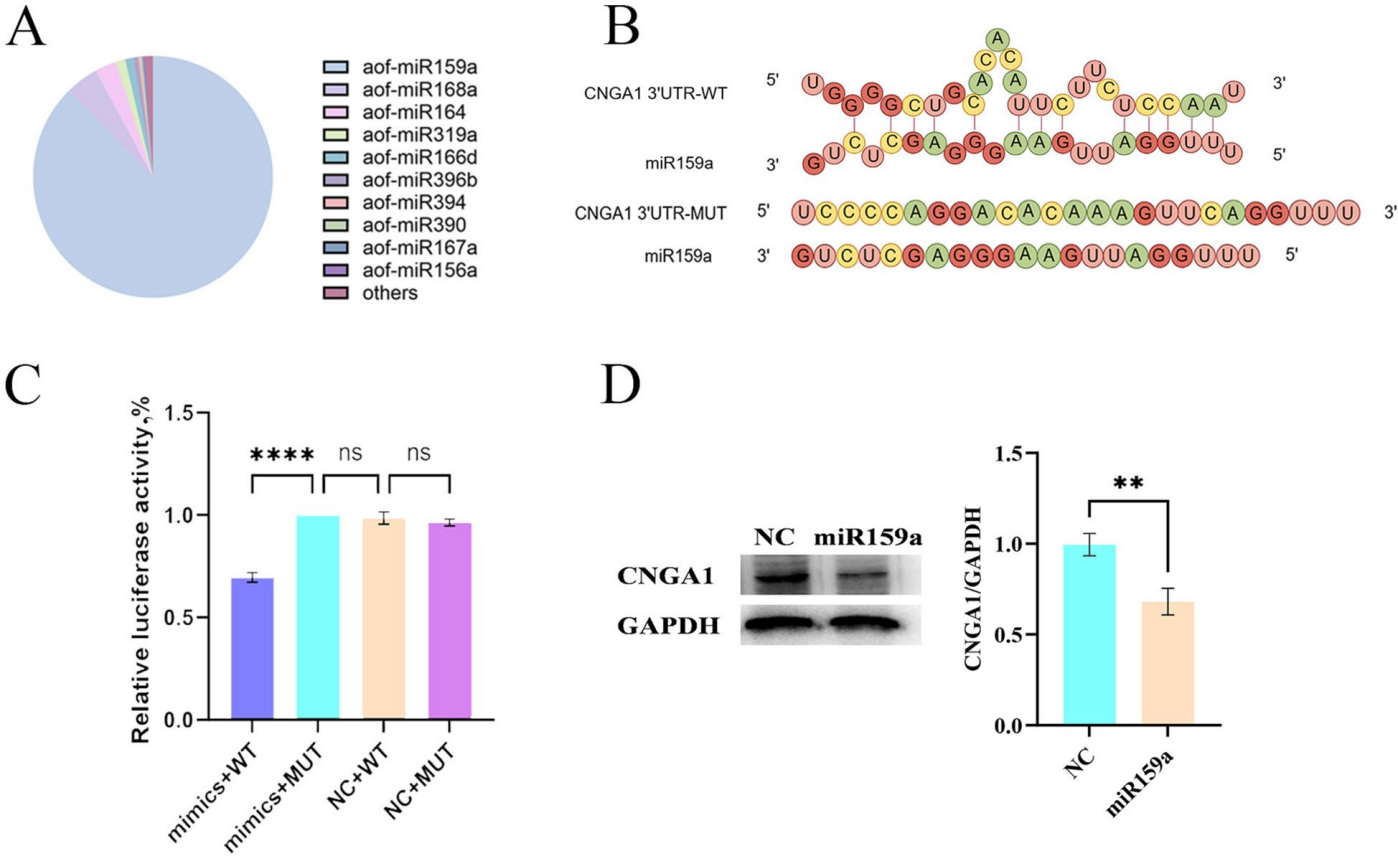
miR159a inhibits the expression of CNGA1 protein. **A** miRNAs extraction and quantification. **B** miR159a target gene prediction and mutation site design. **C** The verification results of the dual-luciferase assay. **D** CNGA1 protein expression levels and quantification. ^**^P < 0.01, ^****^P < 0.0001

To investigate the mechanism underlying the anti-inflammatory effects of SBELNs, the primary miRNA constituents of SBELNs were isolated and characterized. The results showed that SBELNs contain a large amount of miR159a, accounting for 90% of the total miRNAs. Therefore, miR159a may be the key to the function of SBELNs (Fig. 5A). To verify this view, we further screened the target genes of miR159a using the whole chicken genome as a template. Through the prediction results of Mirnada, a total of 6 target genes that met the requirements were obtained. The top three genes with higher scores were analyzed using RNAhybrid. Based on the prediction results of both methods, *CNGA1* was selected as the target gene (Tables 3 and 4). In addition, wild type and mutant type double luciferase reporter expression vectors containing the 3'UTR of *CNGA1* were constructed to validate the relationship between miRNA and target genes (Fig. 5B). After co-transfecting miR159a mimics and the reporter plasmid into 293 T cells, it was found that the luciferase activity in the mimics + WT group was significantly inhibited compared with the NC group (Fig. 5C). In addition, the expression level of CNGA1 protein in HD-11 cells after transfection with miR159a was further detected, and the results showed that miR159a reduced the expression level of CNGA1 protein in HD-11 cells (Fig. 5D). In conclusion, these findings confirmed that miR159a contained in SBELNs can specifically bind to the 3′UTR of *CNGA1* mRNA in HD-11 cells, inhibiting the translation of *CNGA1* mRNA into protein and ultimately reducing the expression level of CNGA1 protein.

**Table 3:** Mirnada target gene prediction results

miRNA	Target	TotScore	TotEnergy	Strand	miRNALen	TargetLen	Positions
aof-miR159a	AGMO	186	−32.72	322	21	1,293	948
aof-miR159a	CNGA1	171	−31.6	2,353	21	1938	577
aof-miR159a	DOLPP1	163	−30.4	3,235	21	738	620
aof-miR159a	PCARE	163	−30.46	11,841	21	3,810	2,686
aof-miR159a	ZRSR2	147	−31.81	16,912	21	1,437	1,354
aof-miR159a	LYST	141	−30.17	10,085	21	11,382	5,293

**Table 4:** RNAhybrid target gene prediction results

miRNA	Target	TotEnergy	miRNALen	TargetLen	Positions
aof-miR159a	AGMO	−34.0	21	1,293	948
aof-miR159a	CNGA1	−34.5	21	1938	577
aof-miR159a	DOLPP1	−32.5	21	738	620

### The potential mechanism of action of miR159a

**Fig. 6:**
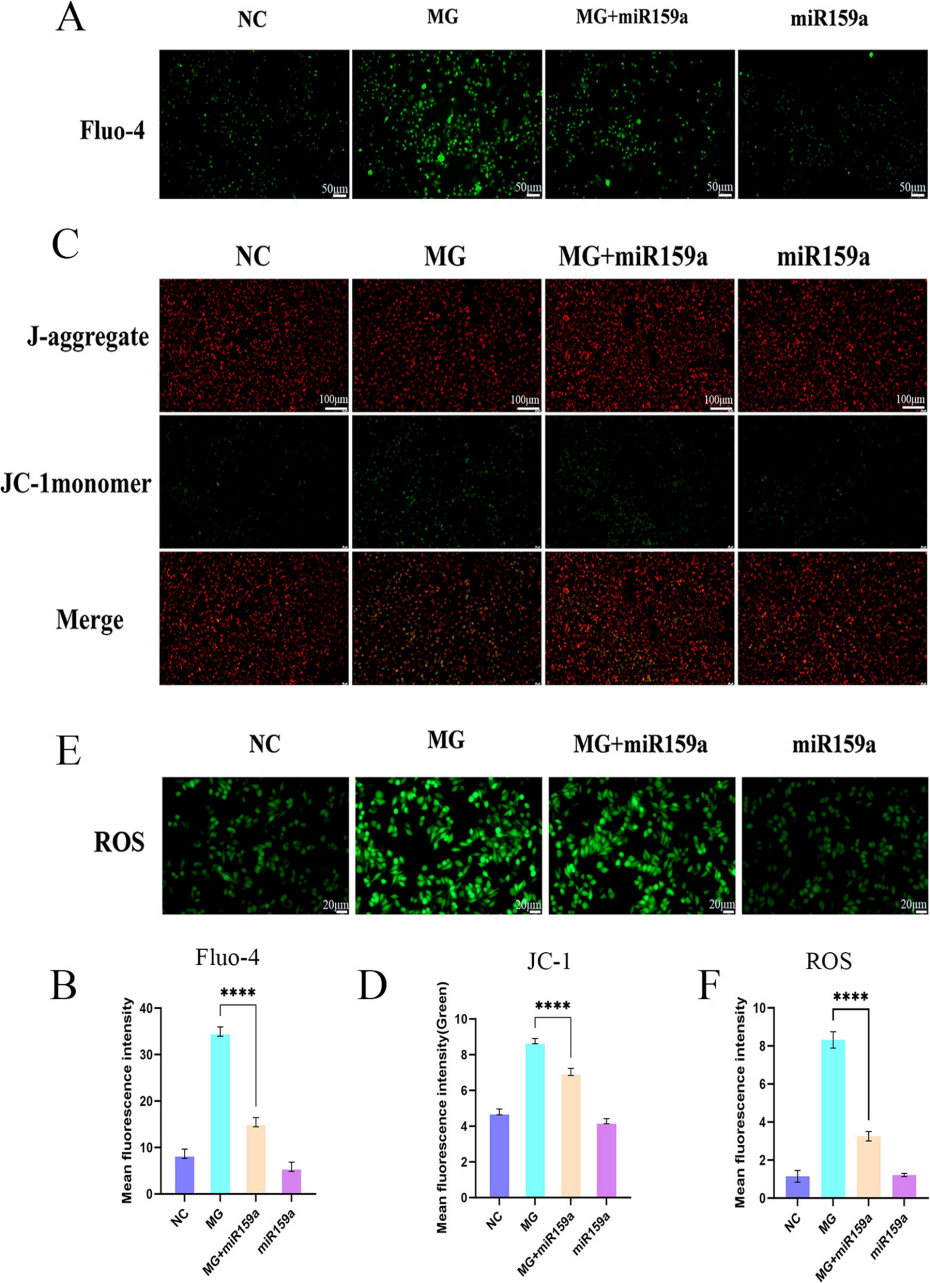
The potential mechanism of action of miR159a. **A** The cytoplasmic calcium ions of HD-11 cells were stained with the fluorescent dye Fluo-4-AM. **C** The mitochondrial membrane potential of HD-11 was detected using the JC-1 detection kit: under normal conditions, the green light emitted by mitochondria is weak, but it will intensify when they are damaged; **E** The effect of miR159a on the production of ROS in MG-infected HD-11 cell. **B**, **D**, and **F** The quantification results of the mean optical density (MOD) of each sample were presented. The results were quantified by analyzing the ratio of the fluorescence density (green) of individual cells to the area of individual cells (*n* = 30). The data are presented as the mean ± standard deviation (SD) ^****^P < 0.0001 vs. MG

CNGA1 is a calcium ion regulatory protein on the cytomembrane. To investigate the regulatory role of miR159a on calcium ions following its targeted binding to CNGA1, changes in intracellular calcium ion concentrations were measured using the Fluo-4 calcium assay kit. Compared with the NC group, the intracellular calcium ions would significantly increase after the cells were treated with MG. However, the intervention of miR159a could obviously reduce the elevation of intracellular calcium ions. The addition of miR159a alone had no significant effect on the calcium ion levels in normal cells (Fig. 6A and B). An elevation in intracellular calcium ion concentration can subsequently impact mitochondrial calcium ion homeostasis via multiple pathways [22]. The results of the JC-1 mitochondrial membrane potential detection kit showed that compared with the NC group, the intensity of green fluorescence in the cells of MG group was significantly enhanced, indicating that MG action on cells can cause mitochondrial membrane potential depolarization; under the intervention of miR159a, the intensity of green fluorescence decreased, and the mitochondrial membrane potential was partially restored (Fig. 6C and D). In addition, the ROS detection kit was employed to further determine the damage to mitochondria. The results indicated a significant increase in intracellular ROS levels in MG-infected HD-11 cells compared to the NC group. However, following miR159a intervention, ROS content was markedly reduced (Fig. 6E and F). In conclusion, miR159a can alleviate intracellular calcium overload and mitochondrial damage caused by MG infection by targeting and binding to CNGA1.

### miR159a alleviates cellular inflammatory damage

**Fig. 7:**
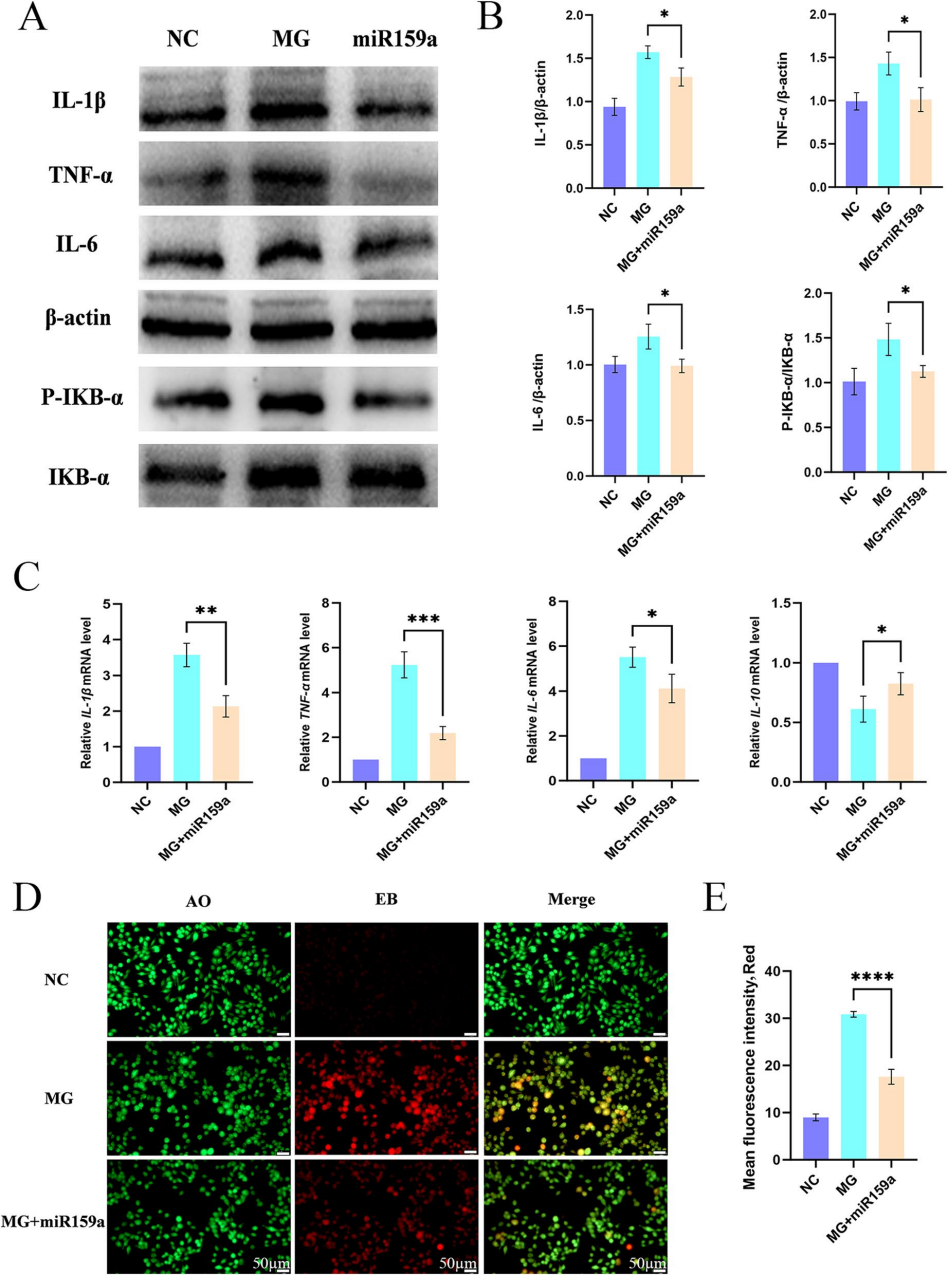
miR159a alleviate cellular inflammatory injury. **A** and **B** WB detection of inflammatory indicators and quantification. **C** qRT-PCR detection of inflammatory indicators. **D** and **E** AO-EB kit detection of cellular injury: normal cells are green and spindle-shaped, while damaged cells are oval and pale yellow. ^*^P < 0.05, ^**^P < 0.01, ^***^P < 0.001, ^****^P < 0.0001

MG infection can lead to calcium overload and mitochondrial damage. Then the excessive intracellular calcium influx and the generation of ROS can both induce an inflammatory response. Therefore, we once again investigated the effect of MG infection on the expression of intracellular inflammatory factors under the intervention of miR159a. The results of WB detection showed that after MG infection, the expression of pro-inflammatory factors such as IL-1β, TNF-α, IL-6, and P-IKB-α/IKB-α in HD-11 cells significantly increased, and the expression levels of inflammatory factors were partially restored after the addition of miR159a (Fig. 7A and B). The results of qRT-PCR detection showed that after MG infection, the expression of pro-inflammatory factors such as *IL-1β*, *TNF-α*, and *IL-6* in HD-11 cells significantly increased, while the expression of the anti-inflammatory factor *IL-10* decreased. After the addition of miR159a, the expression levels of inflammatory factors were partially restored (Fig. 7C). The results of AO-EB kit detection showed that after MG infection, the intensity of red fluorescence in cells significantly increased, and a large number of cells were damaged. After the addition of miR159a, the intensity of red fluorescence partially recovered, and cell damage was alleviated (Fig. 7D and E). In summary, miR159a can alleviate the inflammatory damage caused by MG infection.

## Discussion

ELNs function as mediators of intercellular communication. Recent research has revealed that PDELNs can be internalized by animal organisms, thereby enhancing immune responses and combating infections [23]. In the realm of TCM research, ELNs typically incorporate the bioactive components present in foods. These components can reach the host organism and actively engage in modulating the immune system of the host [24]. In this study, SBELNs were extracted from the roots of fresh *Scutellaria baicalensis* via the ultracentrifugation method. Subsequently, various analytical methods were employed to identify the components of SBELNs. LC–MS/MS analysis revealed that SBELNs contain a variety of flavonoids, such as baicalin and wogonin. Most of these flavonoids exhibit anti-inflammatory and anti-infective activities [25]. This result not only verified the safety of *Scutellaria baicalensis* as a TCM but also laid a solid foundation for the subsequent exploration of SBELNs in disease treatment.

Through extensive practice in the field of TCM, *Scutellaria baicalensis* has been observed to have a significant therapeutic effect on lung diseases [8]. To explore the potential mechanism of *Scutellaria baicalensis* in treating pulmonary diseases, the systematic studies were conducted on the mechanism of *Scutellaria baicalensis* in treating MG infection at the level of ELNs. Intriguingly, the findings indicated that after intragastric administration, the SBELNs absorbed by the digestive tract could specifically accumulate in the lung tissue. This targeting property may be ascribed to the specific proteins or lipid components present on SBELNs surface. Plant-derived exosome-like nanoparticles possess natural targeting properties, mainly due to the plant-specific biomolecules such as proteins, lipids and small RNAs carried on their surface, which can recognize and preferentially bind to specific animal organs through receptor-ligand interactions. Their lipid membranes are rich in plant lipids such as phosphatidic acid, which can enhance accumulation and cellular uptake in target tissues. PDELNs from different sources enter cells through differential endocytic mechanisms, thereby achieving tissue-specific delivery and regulation [26]. Research has demonstrated that integrins are crucial in facilitating the metastasis of tumor-derived exosomes. These proteins specifically direct organotropic metastasis by enhancing exosome adhesion to target cells and increasing vascular permeability [27]. Compared to conventional drug delivery systems, ELNs exhibit inherently low immunogenicity and possess the capability to traverse biological barriers, rendering them especially suitable for the targeted therapy of specific diseases [28]. This property provides support for innovative applications of PDELNs in drug delivery systems.

To further investigate the effects of SBELNs in lung tissue during MG-induced pulmonary infection, inflammation-related markers were assessed at both the tissue and cellular levels. The results showed that SBELNs significantly alleviated alveolar structural damage and reduced inflammation caused by MG infection. This effect may be associated with its multiple regulatory mechanisms: on one hand, the presence of flavonoids, including *baicalein* and *wogonin*, in SBELNs can directly impede MG proliferation [29]. On the other hand, SBELNs exert protective effects indirectly by modulating the host immune response and cellular homeostasis [30]. It is worth mentioning that the therapeutic efficacy of SBELNs may not be attributable to a single component but rather to the synergistic action of multiple active molecules, such as miRNAs and metabolites. Then, we further detected the miRNA in SBELNs and further explored the significant role of miRNA in resisting MG infection.

As key second messengers, calcium ions play an essential role in maintaining intracellular homeostasis. Studies have shown that MG infection significantly upregulates stromal interaction molecule 1 expression, leading to calcium overload and oxidative stress [31]. In this study, it is found that calcium overload induced by MG triggers a pathological cascade involving mitochondrial dysfunction, excessive ROS production, and NF-κB -mediated inflammation. Therefore, effectively regulating the substantial influx of calcium ions may represent a critical approach in the treatment of MG infection. It was identified that SBELNs enter HD-11 cells and subsequently release miR159a. Interestingly, miR159a targets and downregulates the mRNA expression of the calcium ion channel protein CNGA1 in the cell membrane [32], thereby mitigating the excessive influx of calcium ions induced by MG and restoring intracellular calcium homeostasis. Our result bears resemblance to previous studies, the upregulation of miR-21 induced by infection can target and modulate SATB1 and S100 calcium-binding protein A9, thereby regulating intracellular calcium ion concentrations. This regulation subsequently influences the expression of inflammatory factors and plays an important role in disease progression [33]. This study focuses on the targeting relationship between miR159a and CNGA1, and correlates this interaction with MG infection HD-11 cell model. This research offers novel insights into the immunomodulatory functions of TCM-derived ELNs.

Mitochondria serve as the central hub for cellular energy metabolism, primarily responsible for ATP production. Calcium ions play a significant role in regulating mitochondrial function [34]. Within the normal concentration range, calcium ions can enhance ATP synthesis and promote energy production in mitochondria. However, excessive accumulation of calcium ions within mitochondria leads to increased ROS generation, triggers the opening of the permeability transition pore, and subsequently results in ROS outbreak and cellular inflammatory responses [35]. Given that MG infection can lead to a significant influx of calcium ions into cells, we speculated that mitochondrial function might also be compromised. To further evaluate the effect of MG infection on mitochondria, JC-1 staining and ROS detection kits were used. As expected, the disruption of mitochondrial membrane potential and excessive ROS production induced by MG were both alleviated following miR159a introduction. Furthermore‌, mitochondrial ROS are not merely metabolic by-products but also play a key role in cellular signal transduction [36]. An appropriate concentration of ROS can function as signaling molecules to modulate cellular physiological processes, whereas excessive ROS can induce trigger inflammatory responses and cellular damage [37]. Moreover, calcium ions exhibit a strong correlation with the NF-κB inflammatory pathway and can synergistically interact with elevated ROS levels to regulate the release of inflammatory factors [22]. In this study, WB, qRT-PCR and AO-EB staining kits were employed to assess the inflammatory response of cells. The results consistently demonstrated that MG infection leads to an increase in the expression levels of inflammatory factors, while the intervention of miR159a effectively attenuate the expression of these factors.

This study demonstrated that SBELNs display a remarkable tropism for lung tissue. Subsequently, they release bioactive components, thus mitigating the lung inflammatory damage triggered by MG infection. This effect is attributed to two factors. First, metabolites encapsulated in SBELNs directly inhibit MG proliferation. Second, MG infection induces calcium overload, which triggers a cascade of inflammatory responses and lung tissue damage. Notably, miR159a encapsulated within SBELNs targets and binds to the cell membrane-expressed calcium ion channel protein CNGA1, effectively attenuating calcium ion influx. This mechanism indirectly alleviates the inflammatory damage caused by MG. This multi-level regulatory network underscores the "multi-target, multi-pathway" characteristics of SBELNs in MG infection, highlighting the systemic regulatory advantages of TCM components.

However, certain limitations exist in this study. The pathological process of MG infection involves complex interactions among the host, pathogen and immune system, while this study primarily focuses on calcium homeostasis regulation in HD-11. Future research is obligated to integrate the functions of other immune cells, such as T cells and epithelial cells. In addition, the specific targeting relationship between miR159a and CNGA1 was only verified by dual-luciferase reporter assay and protein expression levels. Future experiments can further verify this by knocking out the expression level of CNGA1 in HD-11 cells through molecular techniques. Moreover, it remains ambiguous whether SBELNs encompass other regulatory molecules, such as anti-inflammatory proteins or lipid mediators apart from miRNA and metabolite, thereby demanding in-depth analysis through the combination of proteomics and metabolomics. At the last, research regarding the role of mitochondria in the disease is currently at the stage of characterization, which requires further investigations to clarify its specific mechanisms.

## Supplementary Information

Additional file 1. Raw Western blot images.

## Data Availability

The datasets used and analyzed during the current study are available from the corresponding author on reasonable request.

## Abbreviations

AO: Acridine Orange
AFM: Atomic force microscope
CNGA1: Cyclic nucleotide-gated channel alpha 1
DAPI: 4',6-Diamidino-2-phenylindole
EB: Ethidium Bromide
ELNs: Exosome-like nanoparticles
HD-11: Chicken-like macrophages
HE: Histopathological examination
LC–MS/MS: Liquid chromatography tandem mass spectrometry
IL-1β: Interleukin-1β
IL-6: Interleukin-6
IL-10: Interleukin 10
MG: *Mycoplasma gallisepticum*
MOD: Mean optical density
MicroRNA159a: miR159a
MUT: Mutant type
NTA: Nanoparticle tracking analysis
NF-κB: Nuclear factor-κB
PBS: Phosphate Buffer Saline
PDELNs: Plant-derived exosome-like nanoparticles
ROS: Reactive oxygen species
TCM: Traditional Chinese medicine
TEM: Transmission electron microscopy
TNF-α: Tumor necrosis factor-alpha
UTR: Untranslated regions
WB: Western blotting
WT: Wild type
