# Supplementary figures and images for: Scutellaria baicalensis exosome-like nanoparticles combat lung infection caused by Mycoplasma gallisepticum by regulating calcium homeostasis
Source: J Anim Sci Biotechnol. 2026 May 5;17:83. doi: 10.1186/s40104-026-01395-x (PMC13141375; doi:10.1186/s40104-026-01395-x)

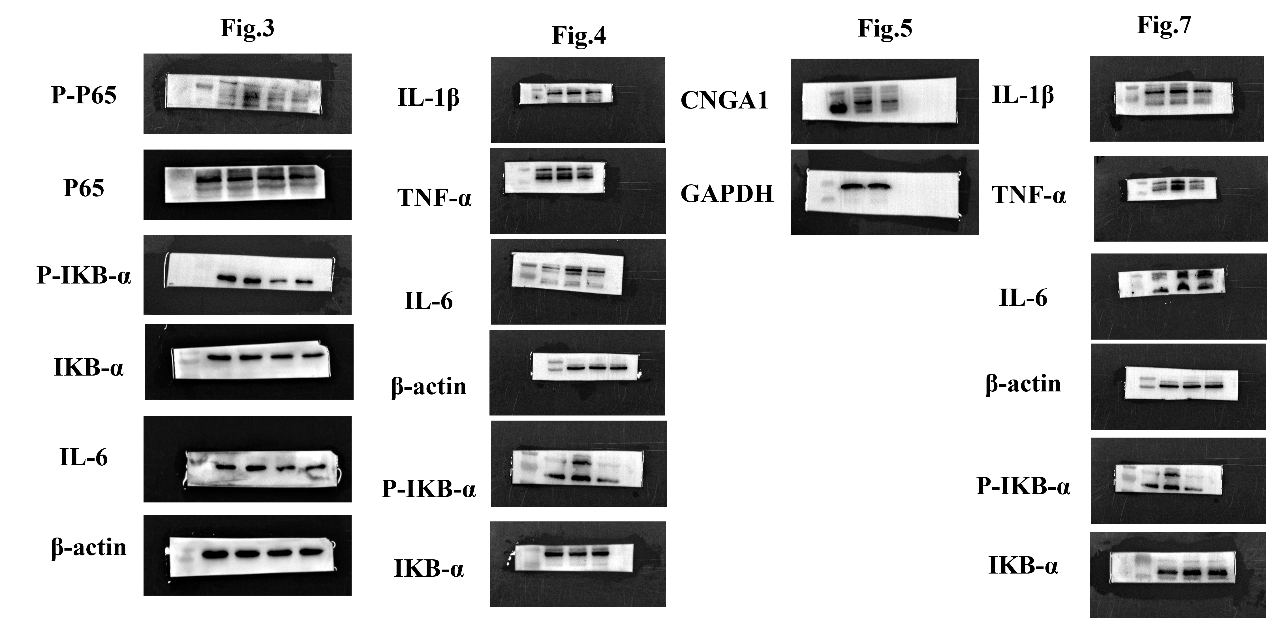

Supplement: Supplementary file 1 — Additional file 1. Raw Western blot images. [file 40104_2026_1395_MOESM1_ESM.docx]
